# Supplementary figures and images for: Potency of bisresorcinol from Heliciopsis terminalis on skin aging: in vitro bioactivities and molecular interactions
Source: PeerJ. 2021 Jun 22;9:e11618. doi: 10.7717/peerj.11618 (PMC8231342; doi:10.7717/peerj.11618)

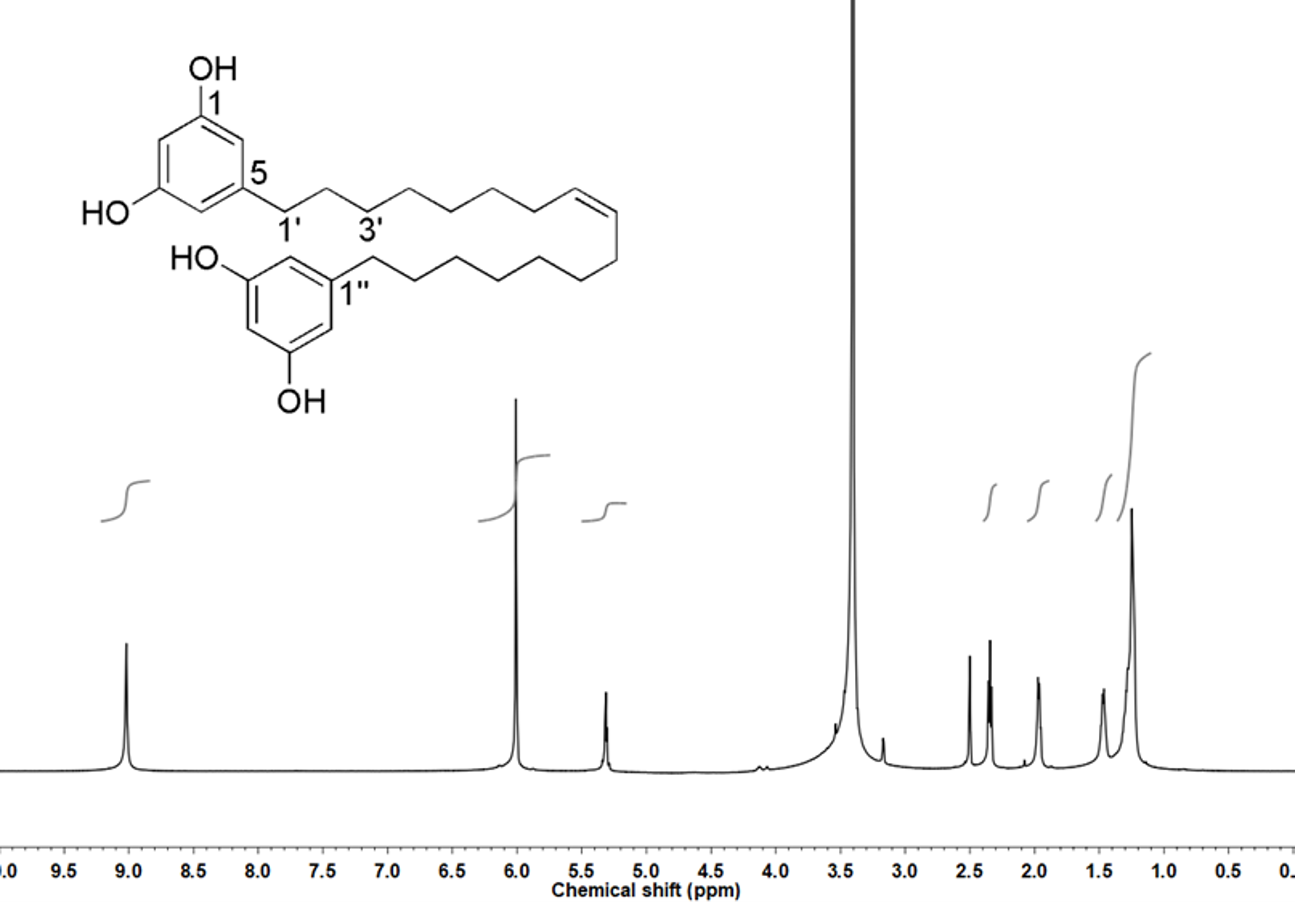

Supplement: Supplemental Information 1 [file peerj-09-11618-s001.png]

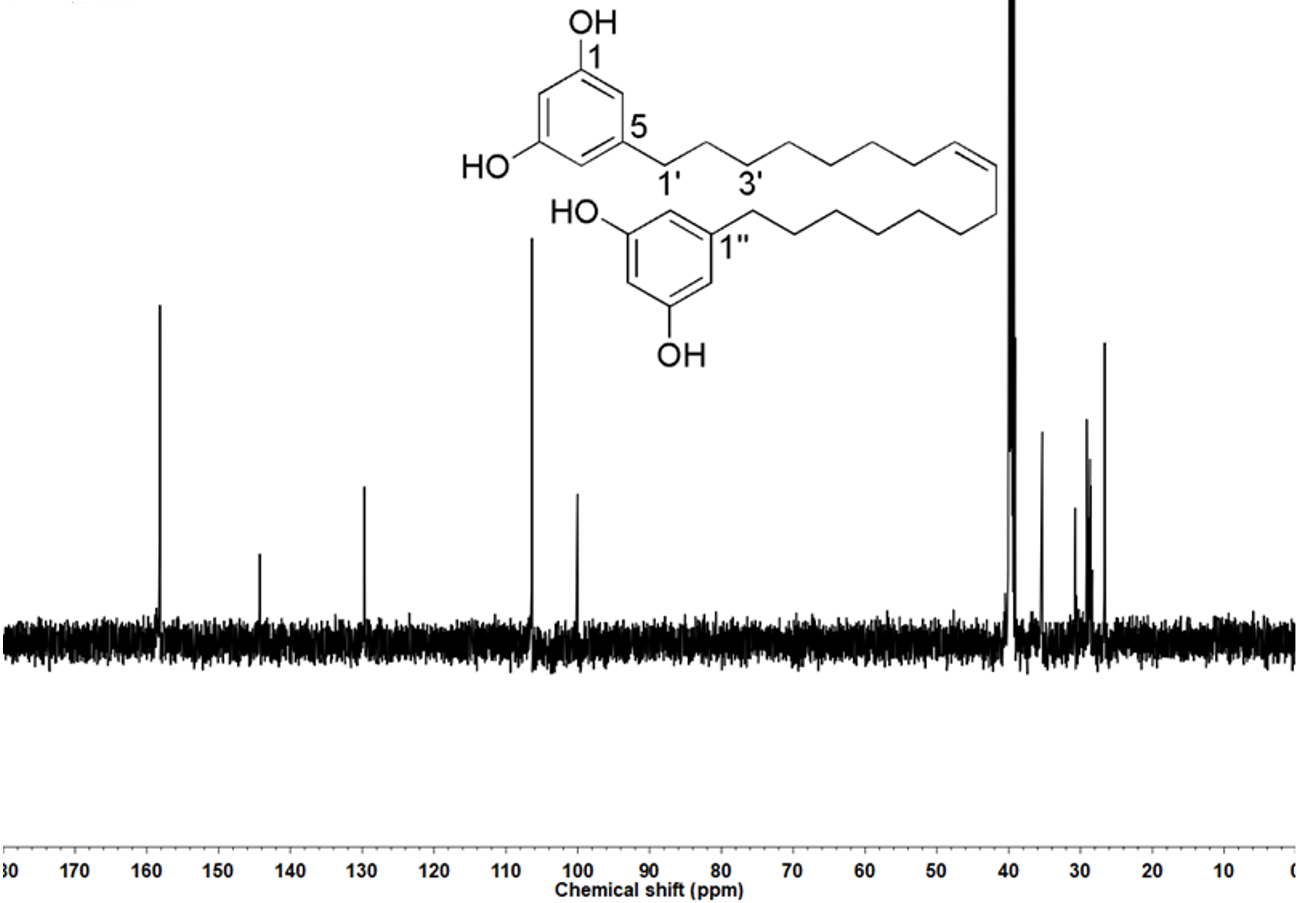

Supplement: Supplemental Information 2 [file peerj-09-11618-s002.png]
